# HIRSUTISM: EVALUATION AND TREATMENT

**DOI:** 10.4103/0019-5154.60342

**Published:** 2010

**Authors:** Silonie Sachdeva

**Affiliations:** *From the Department of Dermatology, Carolena Skin, Laser & Research Centre, Jalandhar - 144022 Punjab, India.*

**Keywords:** *Hirsutism*, *evaluation*, *treatment*

## Abstract

Hirsutism is a common clinical condition seen in female patients of all ages. It affects around 5-10% of the women and is a common presenting complaint in the dermatological out patient department for cosmetic reasons. The cause is mainly hyperandrogeneism, which may be ovarian or adrenal. It may be part of a rare metabolic syndrome, drug induced, or just idiopathic. Hirsutism has a huge psychosocial impact, especially in the young females. This article reviews the current evaluation guidelines and management of hirsutism.

## Introduction

Hirsutism is defined as the presence of terminal coarse hairs in females in a male-like distribution. It affects around 5-10% of women[1,2] and is a common presenting complaint in the dermatological out patient department (OPD) for cosmetic reasons. It is not only imperative to identify the cause of hirsutism but also important to know how to recommend the right treatment based on the main causative factor. The most important determinant in making the diagnosis is a change in the form and rate of hair growth. A technique has been developed to assess hirsutism with video equipment and computer software.[3] Digital imaging of hair development is recorded, which demonstrates a significant difference in hair form and growth rate between hirsute and non-hirsute women.

### Etiology

**Figure 1:**
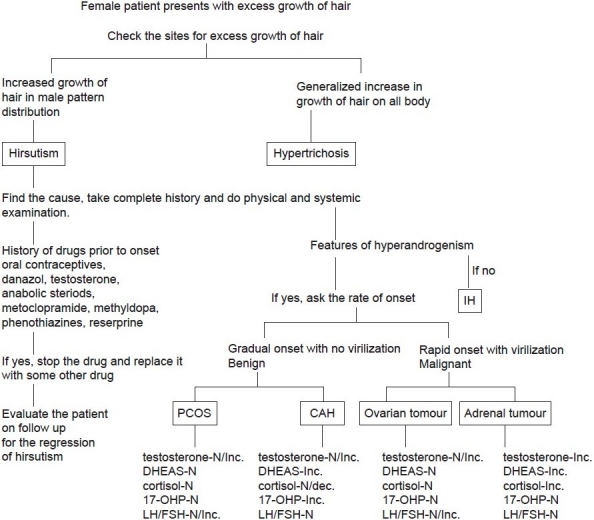
Algorithim showing evaluation of a female patient with excess growth of hair (IH- Idipathic hirsutism, CAH- Congenital adrenal hyperplasia, PCOS- Polycystic ovarian syndrome, DHEAS- Dehydroepiandrosterone sulfate, 17 OHP- 17 Hydroxy progesterone, LH- Luteinizing Hormone, FSH- Follicle stimulating Hormone, (+) present, (−) absent,, N- Normal, Inc.- Increased, dec.- Decreased)

Classically, hirsutism has been considered a marker of increased androgen levels in females from increased production of androgens (i.e testosterone) either by the adrenals or due to an ovarian disease.[4,5] The ovarian causes for hyperandrogenism are polycystic ovarian syndrome (PCOS) and ovarian tumors. Adrenal causes include Cushing's syndrome, androgen-producing tumors, and congenital adrenal hyperplasia (CAH), most commonly due to 21-hydroxylase deficiency. Less common causes include the hyperandrogenic-insulin resistant-acanthosis nigricans syndrome (HAIRAN). Hyperprolactinemia by increasing adrenal dihydroepiandrosterone sulfate (DHEA-S) production may cause hirsutism. Androgenic drugs are also an important cause of hirsutism[6] [Figure 1]. About 20% of the patients may present with idiopathic hirsutism (IH) with normal androgen levels and ovarian function. The cause of increased hair in these women is thought to be related to disorders in peripheral androgen activity.[7] Onset of IH occurs shortly after puberty with slow progression. PCOS and IH account for 90% of the hirstutism in women. Hirsutism can also occur in some premenopausal women and continue for a few years after menopause. This is due to decrease in ovarian estrogen secretion with continuous androgen production.[8]

### Pathogenesis

Hirsutism is attributed either to increased production or increased sensitivity of the hair follicles to circulating androgen (testosterone, Tst). Majority of Tst is secreted either by the ovaries or adrenals (80%). A small amount of circulating Tst is derived from the conversion of androgenic precursors, mainly androstenedione (derived from the ovaries and adrenals) and dihydroepiandrosterone (DHEA—derived from the adrenals) in liver, skin and adipose tissue. However, only 1-2% of Tst is in free form and is the active androgen. About 98-99% is bound to steroid hormone binding globulin (SHBG), cortisol binding globulin, or nonspecifically to albumin and other proteins and is biologically inactive. Only free Tst is converted to dihydrotestosterone (DHT), by the enzyme 5-alpha reductase type 2 isoenzyme present in the outer root sheath of the hair follicles.[4,5] This isoenzyme predominates in the testes, prostate, and the hair follicles of beard and genital hair DHT causes terminalisation of the vellus hair and prolongs the anagen phase resulting in longer thicker hairs. IH with normal androgen levels is postulated to result from exaggerated peripheral 5-alpha reductase activity, androgen receptor polymorphisms, or altered androgen metabolism.[7]

### Clincal features

**Figure 2:**
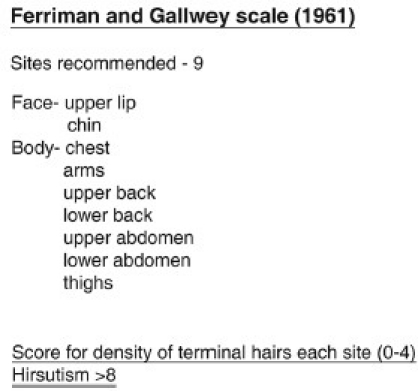
Ferriman and Gallwey scale for measurement of hirsutism[2]

Hirsuate women usually present with increased growth of terminal hair at sides of the face, upper lip, chin, upper back, shoulders, sternum, and upper abdomen. Ferriman and Gallwey[2] devised a score for clinical quantification of hirsutism. In their study of 161 women aged 18 to 38 years, they graded density of terminal hair at nine different body sites under androgen effect from 0 (absence of terminal hairs) through 4 (extensive terminal hair growth) and concluded that hirsutism was represented by a score of 8 or more [Figure 2]. However, this is a subjective scale and hence not universally adopted.

### Evaluation of hirsute patients

When a female patient presents with the chief complaint of increased growth of hair, it is imperative to see whether the coarse hairs are localized in male distribution or there is generalized increase in growth of the hair on all of the body (hypertrichosis).[9,10] After determining hirsutism, the follwing steps can be undertaken to find the cause [Figure 1].

1. Detailed history incuding age of onset (puberty, middle age, menopause), rate of onset of symptoms (gradual or sudden), any signs or symptoms of virilisation (acne, deepening of voice, infrequent mensturation, loss of breast tissue or loss of normal female body contour, clitoromegaly, increased libido, increased muscle mass as in shoulder girdle, malodorous perspiration etc), history of weight gain or diabetes and drug history prior to onset should be taken.
2. Complete general physical and systemic examination should be done including palpation of abdomen for any ovarian mass.
3. If drug is the cause, simple withdrawal of the drug should be helpful. For all other cases, laboratory evaluation of the serum markers to know the exact etiology should be done. The various serum markers are-
  - Testosterone - Serum testosterone may be normal to increased in case of benign pathology as PCOS and CAH but would be definitely raised (>200 ng/ml) in case of malignant tumor of the adrenal or ovary.[11]
  - Dehydroepiandrosterone sulfate (DHEAS) - Raised DHEAS (>700 μg/dl) always indicates an adrenal cause, benign or malignant.
  - 17 Hydroxy progesterone - This serum marker is unique for congenital adrenal hyperplasia. The measurement should be done between 0700 and 0900 hours in the early follicular phase of the menstrual cycle. Levels less than 200 ng/dl excludes the disease. Mildly increased levels between 300 and 1,000 ng/dl require an ACTH stimulation test. Cosyntropin (synthetic ACTH), 250 μg, is administered intravenously, and levels of 17-hydroxyprogesterone are measured before and one hour after the injection. Post-stimulation values (>1,000 ng/dl) constitute a positive test.[11]
  - Twenty four hour urine free cortisol should be measured in women with signs and symptoms of Cushing's syndrome.
  - LH/FSH greater than 3 is indicative of PCOS.[12]
  - Prolactin would be raised in hyperprolactinemia due to hypothalamic disease or a pituitary tumor.
  - Serum TSH: Hypophyseal hypothyroidism[13] can act as a cofactor in hirsutism causing raised TSH.
4. Pelvic ultrasonography can be done to detect an ovarian neoplasm or a polycystic ovary.
5. Magnetic resonance imaging (MRI) or computed tomography (*CT*) of the adrenal region is useful for diagnosis.

## Treatment

Most women resort to removal of hair by different epilation methods, such as plucking, shaving, and waxing before presenting to the clinic. Though simple and inexpensive, these methods are temporary and have their own side effects like physical discomfort, scarring, folliculitis, irritant dermatitis or discoloration. Electrolysis has also been used for the removal of the hair. With repeated treatments, the efficacy ranges from 15 to 50% permanent hair loss.[14] However, it is difficult to treat large areas like hairs on the chest or upper back with electrolysis and it can be time consuming.

Lasers have gained wide popularity in past two decades and can achieve permanent reduction of hair (not removal). They work on the principle of selective photothermolysis where the laser energy acts specifically to destroy the target (melanin).[15,16] Laser energy acts on only anagen hair follicles. Therefore, multiple treatment is required to get a significant (i.e. 80%) reduction. An ideal candidate for laser hair removal is a patient with light skin color and dark colored hairs. Different lasers for hair removal include 694-nm ruby laser, the 1064-nm Q-switched Nd: YAG laser, the 755 nm long-pulsed alexandrite, and the 800-nm diode laser. For Indian skin types (Type IV and V), long wavelength lasers like the Nd Yag laser have been found to be most effective.[17] Laser hair removal is most suitable for idiopathic hirsutism with the normal androgen levels.

### Treatment with drugs

Before starting medicine, diet and exercise should be advised to all women with PCOS. For all obese women, weight loss as a therapy should be advised. Upper body obesity has been shown to be associated with a reduced sex hormone-binding globulin level and increased free Tst levels in both non-hirsute and hirsute women and can contribute to hirsutism. Drugs are indicated for treatment when hyperandrogenism is confirmed by various laboratory tests. The following drugs can be used:

#### Oral contraceptives

Oral contraceptives (OCP) are first-line treatment for hirsutism, particularly in those women desiring contraception. Estrogen/progesterone combinations act by-

- reducing gonadotropin secretion and thereby reducing ovarian androgen production.[18]
- increasing levels of SHBG resulting in lower levels of free testosterone.
- inhibiting adrenal androgen production.[19]

#### Androgen receptor blockers

1. Spironolactone (SPA) is an androgen blocker and competes with DHT for binding to the androgen receptor. SPA also has variable progestational activity and decreases production of ovarian androgens. SPA has an inhibitory effect on 5 alpha-reductase activity (5-RA) and competes with androgens for binding to SHBG. The starting dose is 50 mg twice daily and may be increased to a total daily dose of 200 mg. It takes at least six months to have any beneficial effect. The use of SPA is recommended with the OCP which provides adequate contraception and also helps to minimize the dysfunctional uterine bleeding.[19] Side effects include polyuria, and hypotension with associated headaches, fatigue, or even syncope. SPA should not be used in conjunction with other potassium-sparing diuretics, thiazides, in renal insufficiency, or with excess potassium intake, since patients may develop life-threatening hyperkalemia. It is recommended that serum electrolytes and blood pressure be evaluated two to four weeks after treatment is started. Other minor side effects commonly associated with SPA use include gastritis/dyspepsia and dry skin; SPA should be taken with food as this increases its absorption and reduces its potential for gastritis.[20] Absolute contraindications to SPA use include renal insufficiency, anuria, chronic renal impairment, hyperkalemia, pregnancy, and abnormal uterine bleeding.
2. Cyproterone Acetate (CA) has strong progestogenic and antiandrogen properties. It produces a decrease in circulating Tst and androstenedione levels through a reduction in circulating LH and has been used as an effective treatment for hirsutism.[21] CPA is available in combination with ethinyl estradiol (EE) (2 mg CPA and 35 μg EE/tablet).

#### 5-RA inhibitors

Finasteride, a 5-alpha reductase inhibtor has been found to be effective in the treatment of IH.[22] Finasteride primarily inhibits type 2 5-RA activity. A 5-RA inhibitor still in clinical testing is dutasteride (GI198745, GlaxoWellcome Co., Research Triangle Park, NC), a “dual” type 1 and type 2 5-RA inhibitor. It is predicted that this drug will be more potent than finasteride. This compound effectively inhibits DHT production by 99% approximately 24 h after oral administration. All these 5-RA agents have the potential of feminizing a male fetus. Hence, effective contraception must be used by patients on these drugs.

#### Gonadotrophin-releasing hormone (GnRH agonists)

This therapy is parenteral and reserved for women with severe hirsutism who don't respond to the OC and antiandrogens.[23] Long-acting GnRH analogs decrease gonadotrophin secretion and therefore reduce ovarian stimulation and hence testosterone. Estrogen production is also reduced. Hence, therapy is usually used in combination with an oral contraceptive pill containing estrogen and progestin.

#### Adrenal suppression: Glucocorticoids

The main use of corticosteroids has been to treat hirsutism associated with congenital adrenal hyperplasia (CAH).[24] They are used in a low bedtime dose of dexamethasone.[25]

#### Biological modifiers of hair follicular growth

Eflornithine hydrochloride is a new agent, which is used as a topical cream (13.9%) for decreasing or arresting facial hair growth in women. It is thought to inhibit hair growth by inhibiting an enzyme involved in keratin synthesis. It is a potent, irreversible inhibitor of the enzyme ornithine decarboxylase, which is necessary for production of the polyamines that mediate cell migration, proliferation, and differentiation.[26] Binding of DHT to the androgen receptor is associated with stimulation of ornithine decarboxylase synthesis and proliferation of hair matrix cells. The cream is applied to the face twice a day. Gradual improvement is seen in six to eight weeks. It can also be used in combination with laser treatments for better effects.[27]

### New drugs in trial

A new topical antiandrogen Fluridil (2-hydroxy-2-methyl-N-[4-nitro-3-(trifluoromethyl) phenyl]-3-(2,2,2-trifluoroacetylamino)ropanamide) has been developed for hyperandrogenic skin syndromes.[28] Based on these results, a study was conducted as an orientational three-month pilot study to evaluate the efficacy and safety of 2% fluridil gel in female patients with idiopathic hirsutism. The present clinical study has shown that a 2% fluridil gel is a safe and effective treatment method of hirsutism. However, this preparation is not available yet. Compared to systemic administration of antiandrogens, topical fluridil does not affect general health and sexual functions and, more importantly, does not decrease libido.

## Conclusion

Hirustism requires indepth clinical evaluation and investigation for treatment. For pharmacological therapy, oral contraceptives is recommended for the majority of women. Adding an antiandrogen may be needed after six months if the response is suboptimal. Antiandrogen monotherapy is not recommended unless adequate contraception is used. For women who choose hair removal therapy, photoeplilation with lasers is the preferred choice.
